# Human biomonitoring of deoxynivalenol (DON) - Assessment of the exposure of young German adults from 1996 - 2021

**DOI:** 10.1016/j.ijheh.2023.114198

**Published:** 2023-07

**Authors:** Andy Schmied, Lennart Marske, Marion Berger, Peter Kujath, Till Weber, Marike Kolossa-Gehring

**Affiliations:** a Federal Institute for Occupational Safety and Health (BAuA), Berlin, Germany; b German Environment Agency (UBA), Berlin, Germany

**Keywords:** Deoxynivalenol, Human biological monitoring (HBM), Environmental specimen bank, HPLC-MS/MS, HBM4EU

## Abstract

The mycotoxin deoxynivalenol (DON) is a frequently found contaminant in cereals and cereal-based products. As a German contribution to the European Joint Programme HBM4EU, we analysed the total DON concentration (tDON) in 24-h urine samples from the German Environmental Specimen Bank (ESB). In total, 360 samples collected in 1996, 2001, 2006, 2011, 2016, and 2021 from young adults in Muenster (Germany), were measured by high performance liquid chromatography-tandem mass spectrometry (HPLC-MS/MS) after enzymatic deconjugation of the glucuronide metabolites.

tDON was found in concentrations above the lower limit of quantification (0.3 μg/L) in 99% of the samples. Medians of the measured concentrations and the daily excretion were 4.3 μg/L and 7.9 μg/24 h, respectively. For only nine participants, urinary tDON concentrations exceeded the provisional Human biomonitoring guidance value (HBM GV) of 23 μg/L.

Urinary tDON concentrations were significantly higher for male participants. However, 24-h excretion values normalized to the participant's body weight did not exhibit any significant difference between males and females and the magnitude remained unchanged over the sampling years with exception of the sampling year 2001.

Daily intakes were estimated from excretion values. Exceedance of the tolerable daily intake (TDI) of 1 μg/kg bw per day was observed for less than 1% of all participants. TDI exceedances were only present in the sampling year 2001 and not in more recent sampling years while exceedance of the HBM guidance value was also observed in 2011 and 2021.

## Highlights

• The mycotoxin DON could be quantified in 99% of all 24h-urine samples.
• Significant higher daily total DON excretion observed for men than for woman.
• Less than 1% of all participants exceed the tolerably daily intake (TDI).
• Magnitude of exposure is largely stable over the sampling period of 25 years.

## 1 Introduction

Deoxynivalenol (DON) is a mycotoxin produced primarily by fungi of the genus *Fusarium*. *Fusarium* fungi belong to the most prevalent toxin-producing fungi infecting cereal crops pre harvest in northern temperate regions (SCF, 2002). Therefore, DON is a frequent contaminant in cereals and cereal-based products. The general population is exposed to this toxin mainly via ingestion of contaminated foods (EFSA Panel on Contaminants in the Food Chain, 2017; Marin et al., 2013). At workplaces, inhalation of bioaerosols and dusts and dermal contact with contaminated material are relevant routes of exposure (Degen, 2011; Fromme et al., 2016; Viegas et al., 2018). General toxicity and immunotoxicity are considered to be critical effects of DON. The Scientific Committee on Food (SCF) of the European Commission derived a tolerable daily intake (TDI) of 1 μg/kg bw per day based on the no observed adverse effect level of 100 μg/kg bw per day and an uncertainty factor of 100 (SCF, 2002). Adverse effects that were taken into account cover growth retardation, increased susceptibility to infections and reproductive effects as most sensitive endpoints observed in animal studies in mice and rats by Iverson et al. (1996), Tryphonas et al. (1986) and Khera et al. (1984), respectively. Since 2017, the TDI is used as group TDI for the sum of DON and co-occurring metabolites of DON (acetylated and modified forms) in food that are largely converted to DON during mammalian digestion (EFSA Panel on Contaminants in the Food Chain, 2017). Based on the TDI, a provisional HBM guidance value of 23 μg DON/L urine was derived for 24-h urine samples (Apel et al., 2023).

Furthermore, in Europe the EU regulation (EC) No 1881/2006 give maximum levels for DON in foodstuffs such as cereals, cereal flour, pasta and bread (European Commission, 2006). DON concentrations exceeding these maximum levels occur and are reported for less than 2% of the analysed foodstuffs samples (EFSA, 2013). In humans, ingested DON is absorbed, distributed, metabolized and excreted to urine rapidly (Mengelers et al., 2019). Approximately 64% of a single oral dose of DON were recovered in human urine within 24 h after administration (Vidal et al., 2018). Conjugation of DON to glucuronic acid is the major metabolic pathway, and DON-15-glucuronide (D15GlcA) is the main conjugation product besides DON-3-glucuronide (D3GlcA) (Warth et al., 2013). In human biomonitoring studies, DON-glucuronides (DGlcA) accounted in average for 66%–91% of the total DON (tDON, sum of free DON plus glucuronides) excreted (Brera et al., 2015; Turner et al., 2011a; Vidal et al., 2018; Warth et al., 2012). De-epoxy-deoxynivalenol (DOM-1) is a further DON metabolite found less frequently in human urine samples (EFSA Panel on Contaminants in the Food Chain, 2017). It is suggested that DOM-1 derives from DON metabolism by intestinal microbiota (Gratz et al., 2013). While tDON correlates well with dietary exposure to DON, DOM-1 is considered to be an unsuitably biomarker of exposure (EFSA Panel on Contaminants in the Food Chain, 2017).

Within the European Human Biomonitoring Initiative (HBM4EU) (Ganzleben et al., 2017; Kolossa-Gehring et al., 2023), a large scale EU project operating at the science-policy interface, substances were systematically prioritized with the aim to harmonise human biomonitoring procedures and collect recent exposure data (Ougier et al., 2021). The resulting highly quality assured and comparable data supply the basis for their use to improve policies and control their effectiveness at national and European scale.

Due to its widespread occurrence and concerns of possible adverse effects on human health, DON has been prioritized in the 2nd HBM4EU prioritisation round and set on the 2nd priority list of substances (HBM4EU, 2018)**.** The combined analytical determination of free DON and DGlcA in urine after enzymatic deconjugation of the glucuronides with *β*-glucuronidase has been chosen and validated as HBM method to assess the DON exposure (Esteban López et al., 2021; Gilles et al., 2022; HBM4EU, 2022). In this study, we applied this method to analyse 360 24-h urine samples from the German Environmental Specimen Bank (ESB) collected from 1996 to 2021. The results provide information on both the recent and the past internal exposure to DON, and hence enable the investigation of the DON exposure over the course of time. Based on the results we estimate the daily intake and assess the exposure level.

## 2 Materials and methods

### 2.1 Study population

In total, 360 24-h urine samples collected from student volunteers in Muenster (containing only one first-morning void) were analysed in this study. The samples were aliquoted, precooled down to −160 °C and then stored under cryogenic conditions over liquid nitrogen in the ESB (Kolossa-Gehring et al., 2012; Lermen et al., 2014).

Per sampling year (1996, 2001, 2006, 2011, 2016, 2021), samples of 60 participants with an equal distribution of males and females were analysed. Table 1 summarizes the characteristics of the study population as well as information on 24-h urine volumes and creatinine contents. All participants provided informed consent. The study protocol of the ESB was approved by the ethics committees of the Medical Association of Westphalia-Lippe (until 2011) and of the Medical Association of the Saarland (since 2011).

**Table 1:** Characteristics of the study population.

Sampling year	N (m/f)	Age [years] AM (range)	bw [kg] AM (range)	24-h urine volume [mL] AM (range)	Creatinine [g/L] AM (range)
1996	60 (30/30)	24.5 (20–29)	68.8 (45–98)	1441 (540–2525)	1.18 (0.49–2.21)
2001	60 (30/30)	23.6 (20–29)	70.1 (44–124)	1698 (510–2800)	1.07 (0.30–2.43)
2006	60 (30/30)	24.0 (20–29)	69.3 (47–112)	1884 (345–4287)	0.91 (0.29–2.52)
2011	60 (30/30)	23.0 (20–29)	71.5 (48–102)	1886 (766–3049)	0.86 (0.25–2.07)
2016	60 (30/30)	23.5 (20–29)	71.5 (48–100)	2122 (569–3207)	0.72 (0.32–2.29)
2021	60 (30/30)	23.0 (20–28)	67.7 (50–94)	2142 (1042–4918)	0.71 (0.26–1.71)
ΣAll	360	23.6 (20–29)	69.8 (44–124)	1862 (345–4918)	0.91 (0.25–2.52)
ΣMale	180	23.9 (20–29)	78.1 (53–124)	1883 (569–4918)	1.07 (0.25–2.43)
ΣFemale	180	23.2 (20–29)	61.5 (44–102)	1841 (345–4410)	0.75 (0.26–2.52)

N: number of samples; m: male; f: female; AM: arithmetic mean; bw: body weight.

### 2.2 Analytical method

Analytical determination of total deoxynivalenol was carried out according to Berger et al. with one modification and involved enzymatic cleavage of glucuronide conjugates with *ß*-glucuronidase from *Escherichia coli* (*E. coli)* followed by immunoaffinity chromatography purification (IAC). Purified and concentrated samples were analysed by HPLC-MS/MS using fully ^13^C-labeled DON as internal standard and a blank urine sample for a matrix-matched calibration that was obtained from a volunteer without consumption of cereals or cereal-based products. The lower limit of quantification (LLOQ) of the applied method was 0.3 μg/L.

DON StarR immunoaffinity columns (Romer Labs, Tulln, Austria) used in our previous study were unavailable. Therefore, DON Star immunoaffinity columns (Romer Labs, Tulln, Austria) were used for IAC purification. While DOM-1 binds to DON StarR (previous study), DON Star columns showed no cross-reactivity for this DON metabolite. No differences were observed for DON.

Creatinine was measured photometrically according to the Jaffé method (Lermen et al., 2014).

### 2.3 Statistical analysis

IBM SPSS Statistics 26 was used for statistical analysis. tDON concentrations below LLOQ were assigned with the value half of the LLOQ. To compare sexes and sampling years, non-parametric tests (Mann-Whitney-*U* test, Kruskal–Wallis test) were applied. Bonferroni-correction was used for post-hoc tests.

Correlations were evaluated using non-parametric Spearman rank correlation analysis. A significance level of α = 0.05 was assigned for statistical significance.

Descriptive statistics are represented as means, percentiles (50th, 90th) and ranges.

Urinary tDON concentrations are given without creatinine adjustment. Daily urinary tDON excretions are shown normalized to the body weight and without normalization.

For latter see supplementary information.

### 2.4 Daily intake estimation

Daily intakes of tDON were estimated from the daily excretion of tDON normalized to the body weight and urinary excretion ratios of 72% and 64% as reported by Turner et al. (2010) and Vidal et al. (2018).

## 3 Results and discussion

### 3.1 Urinary concentrations of tDON

tDON was found in concentrations above the LLOQ (0.3 μg/L) in 357 of the 360 urine samples. Table 2 summarizes the urinary tDON concentrations differentiated for male and female participants and year of sampling.

**Table 2:** Urinary tDON concentrations. Values are given in μg/L.

	Sampling year	1996	2001	2006	2011	2016	2021	Σ1996–2021
All participants	N > LLOQ	60 (100%)	59 (98%)	60 (100%)	60 (100%)	60 (100%)	58 (97%)	357 (99%)
	AM	5.09	13.1	4.90	6.81	4.05	4.97	6.48
	GM	3.85	8.80	3.83	5.37	2.47	3.16	4.19
	Median	3.93	9.11	4.01	5.52	2.26	3.36	4.33
	P90	10.4	26.0	9.79	14.2	10.3	9.37	14.2
	Range	0.45–23.0	<LLOQ–99.1	0.84–18.8	0.38–24.2	0.5–21.3	<LLOQ–24.9	<LLOQ–99.1
Males	N > LLOQ	30 (100%)	29 (97%)	30 (100%)	30 (100%)	30 (100%)	30 (100%)	179 (99%)
	AM	6.19	16.3	5.98	7.25	4.04	7.21	7.51
	GM	4.57	9.88	4.77	5.61	2.56	5.10	5.01
	Median	5.24	11.7	4.78	5.91	2.34	5.52	5.50
	P90	12.6	42.9	10.5	16.0	10.2	17.8	16.50
	Range	0.45–23.0	<LLOQ–99.1	0.92–18.8	1.51–24.2	0.73–19.4	0.73–24.9	<LLOQ–99.1
Females	N > LLOQ	30 (100%)	30 (100%)	30 (100%)	30 (100%)	30 (100%)	28 (93%)	178 (99%)
	AM	3.98	9.83	3.81	6.36	4.06	2.73	5.13
	GM	3.24	7.85	3.07	5.13	2.39	1.97	3.51
	Median	3.33	7.3	2.94	5.03	2.07	2.28	3.54
	P90	7.29	22.6	7.86	13.5	10.6	7.03	11.1
	Range	1.06–19.2	1.72–26.4	0.84–10.4	0.38–16.9	0.5–21.3	<LLOQ–8.66	<LLOQ–26.4

N: number of samples; LLOQ: lower limit of quantification (0.3 μg/L); AM: arithmetic mean; GM: geometric mean; P90: 90th percentile.

For nine of the 360 urine samples, tDON concentrations exceeded the provisional HBM guidance value of 23 μg/L. Most of the exceedances (7 out of 9) were observed in 2001. In 2011 and 2021 only one participant exceeded the guidance value. Male participants had significantly higher urinary tDON concentrations than female participants (Mann-Whitney-*U* test, p < .001). Higher urinary tDON concentrations for males were also reported by Heyndrickx et al. (2015). In contrast, Solfrizzo et al. (2014) observed no differences between the sexes.

After normalization to creatinine, no significant differences between the sexes were observed anymore in our study. This can most probably be explained by significantly higher urinary creatinine concentrations of males in comparison to females (Lermen et al., 2019). The median (maximum) values observed after creatinine adjustment are 5.52 (45.5) μg/g creatinine and 5.54 (29.0) μg/g creatinine for males and females, respectively.

In previous human biomonitoring (HBM) studies (Table 3), urinary DON concentration were determined in first-morning void urine, spot urine and 24-h urine samples. Furthermore, free DON and DON glucuronides were analysed separately as well as combined after enzymatic deconjugation. In the studies of Brera et al. (2015), Solfrizzo et al. (2014) and Xia et al. (2020), children were included additionally in the study population and Shephard et al. (2013) requested their study participants to consume a traditional maize-based evening meal prior urine collection. These differences hamper the comparison of the obtained HBM data. However, the frequency in which DON was observed and the urinary tDON levels found in this study are comparable to previously published data from other European HBM studies and higher compared to levels found in South Asian HBM studies. This observation is in line with the different dietary habits. DON is mainly a contaminant in wheat, barley, maize and products derived thereof whose consumption is lower in South Asia (Ali et al., 2016; Turner et al., 2011b).

**Table 3:** Urinary DON concentrations in human biomonitoring studies.

Country	Collection year(s)	N (m/f)	Study Population	Urine type	Positive, N (%)	Median [μg/L] (range)	Reference
Germany	1996–2021	360 (180/180)	adults	24-h	357 (99)	tDON 4.33 (<0.3–99.1)	This study
Germany	2013	50 (23/27)	adults	1^st^morning	50 (100)	tDON 7.35 (1.06–38.4)	Ali et al. (2016)
Italy	2011	52 (26/26)	children and adults	1^st^morning	50 (96)	tDON 10.3 (<1.5–67.4)	Solfrizzo et al. (2014)
Norway	2014	230 (92/138)	children and adults	1^st^morning	229 (99)	tDON 6.17 (<0.015–86.9)	Brera et al. (2015)
Portugal	2015–2016	94 (48/46)	adults	24-h	59 (63)	DON 2.51 (<1.0–36.3)	Martins et al. (2019)
					35 (37)	D3GlcA <1.0 (<1.0–34.7)	
					48 (51)	D15GlcA 1.73 (<0.9–204)	
Belgium	2013–2014	239 (106/133)	adults	1^st^morning	89 (37)	DON 1.7 (<0.2–130)	Heyndrickx et al. (2015)
					184 (77)	D3GlcA 4.4 (<0.2–126)	
					238 (100)	D15GlcA 31.2 (1.1–461)	
South Africa		53 (0/53)	adults	1^st^morning	53 (100)	tDON 8.95 (<0.5–353)	Shephard et al. (2013)
China	1997–1998	60 (0/60)	adults	spot	58 (97)	tDON 4,8^a^ (<0.5–29.9)	Turner et al. (2011b)
Bangladesh	2013	62 (31/31)	adults	1^st^morning	17 (27)	tDON <0,3 (<0,3–1.78)	Ali et al. (2016)
Pakistan	2014	264 (153/111)	children and adults	spot	54 (20)	tDON <0.5 (<0.5–1.25)	Xia et al. (2020)

N, number of samples; m: male; f: female; 1^st^morning, first-morning void urine sample; ^a^ mean.

### 3.2 Daily excretion values of tDON

**Fig. 1:**
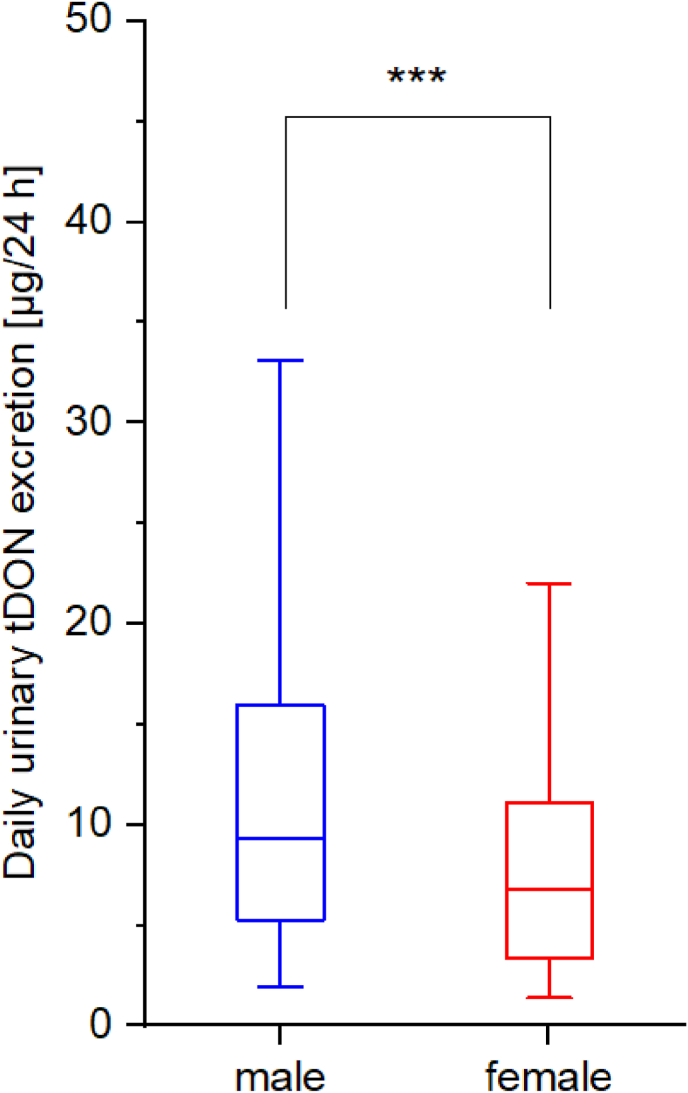
Boxplot of daily urinary tDON excretion. The boxes and boundaries of the whiskers represent the 25th/75th and 5th/95th percentiles, respectively. The horizontal lines indicate the median values. ***: There was a statistically significant difference in daily urinary tDON excretion between the sexes, *p* < .001 (Mann-Whitney-*U* test).

While no differences are observed for the 24-h urine volume (Table 1) of females and males (AM: 1841 *vs*. 1883 mL/24 h; 90th percentiles: 2749 *vs*. 2725 mL/24 h), males exhibit higher urinary tDON concentrations (medians: 3.54 *vs.* 5.50 μg/L; 90th percentiles: 11.1 *vs*. 16.50 μg/L; Table 2). This contributes to a significant higher daily tDON excretion in men than in woman (Mann-Whitney-*U* test, p < .001, Fig. 1). Medians (90th percentile) were 9.30 (23.5) and 6.76 (17.9) μg/24 h, respectively.

However, when daily tDON excretion is normalized to participant's body weight (Table 4) no significant differences are observed between male and female participants (medians: 11.9 *vs*. 11.0 ng/24 h x kg bw; 90th percentiles: 34.5 *vs*. 28.3 ng/24 h x kg bw) indicating an equal magnitude of exposure of both sexes in our study population. Table 4 summarizes the statistics for the daily urinary tDON excretion normalized to body weight.

**Table 4:** Statistics for the daily urinary tDON excretion normalized to body weight. Values are given in ng/kg bw per day.

	Sampling year	1996	2001	2006	2011	2016	2021	Σ1996–2021
All participants	N > LLOQ	60 (100%)	59 (98%)	60 (100%)	60 (100%)	60 (100%)	58 (97%)	357 (99%)
	AM	101	267	116	165	114	136	150
	GM	77	202	98	136	71	96	106
	Median	85	216	111	151	56	103	113
	P90	176	501	205	334	296	297	304
	Range	6–350	6*–1300	15–281	17–548	7–585	3*–529	3*–1300

N: number of samples; LLOQ: lower limit of quantification; AM: arithmetic mean; GM: geometric mean; P90: 90th percentile; *Volume-based DON concentration of the corresponding 24-h urine sample is <LLOQ.

### 3.3 Daily DON intake assessment

One goal of our HBM study was the estimation of the daily DON intake. Urinary excretion ratios of 72.3% (95% CI 59.1–85.5%) and 64.0 ± 22.8% were reported by Turner et al. (2010) and Vidal et al. (2018), respectively. Considering these urinary excretion factors, we estimated the daily DON intake from the daily tDON excretion normalized to participant's body weight. Table 5 summarizes the estimated daily intake for a low level of exposure (10th percentile), an average level of exposure (median) and a high level of exposure (90th percentile). The TDI for DON is 1 μg/kg bw per day. Depending on the excretion factor applied, the estimated daily intake exceeds the TDI for two and three of all participants, respectively (all from sampling year 2001).

**Table 5:** Estimated daily intake of DON, expressed in μg/kg bw per day. P10, 10th percentile; P50, median; P90, 90th percentile; Max, maximum value.

Excretion factor	P10	P50	P90	Max
0.72	0.05	0.16	0.42	1.80
0.64	0.06	0.18	0.47	2.02

In HBM studies from Belgium, Italy, Austria, Portugal and Spain, exceedance of the TDI was observed for 29% (Heyndrickx et al., 2015), 6% (Solfrizzo et al., 2014), 33% (Warth et al., 2012), 10% (Martins et al., 2019) and 8% (Rodríguez-Carrasco et al., 2014) of the participants, respectively. The percentage of TDI exceedance observed in our study is far lower (0.6–0.8%). This observation is in line with the study results of Ali et al. (2016), who did not observe an exceedance of the TDI for a smaller study population from Germany (N = 50). Differences in exposure in different countries could be explained most likely by different dietary habits (Heyndrickx et al., 2015). However, a comparison of the results and the assessment of the DON exposure in the different countries is only possible to a limited extend due to different approaches used to estimate the daily DON intake.

Although a urinary collection period of 24 h is recommended for the estimation of the daily DON intake due to rapid renal DON excretion (Mengelers et al., 2019), first-morning void urine samples were used in the majority of the HBM studies shown here. In addition, different assumptions on the daily urine production and different urinary excretion ratios were applied. Heyndrickx et al. (2015), Martins et al. (2019), and Warth et al. (2012) used an urinary excretion ratio of 72% that was determined by Turner et al. (2010) based on first-morning void urine samples of adults from the United Kingdom. In contrast, Solfrizzo et al. (2014) applied an crudely estimated urinary excretion ratio of 50% (Turner et al., 2008).

As mentioned above, the urinary tDON concentration in this study exceeded the provisional HBM guidance value in nine samples. In fact, sex-specific differences that were observed in this study, the urine volume as well as the body weight are not taken into account if the HBM guidance value is applied. However, all of the samples where the estimated daily intake exceeded the TDI were identified using the HBM guidance value. Thus, the HBM guidance value, that was derived recently, is suited to directly identify body burdens presenting an appreciable risk to health.

### 3.4 Development of tDON levels over time

The ESB study design enables monitoring changes of exposure over time to assess effects or a necessity of policy measures. In previous studies, decreasing trends could be observed e.g. for lead, mercury and phthalates (Bartel-Steinbach et al., 2022; Koch et al., 2017; Lermen et al., 2021). While for other substances like 1,2-Cyclohexane dicarboxylic acid diisononyl ester (DINCH), an alternative for phthalate plasticizers, an increasing trend could be shown (Kasper-Sonnenberg et al., 2019; Lemke et al., 2021).

To assess changes of the DON exposure level, results on tDON were assigned to the respective sampling year. However, a clear time trend could not be observed regardless of the parameter tested.

A weak negative correlation was observed between the urinary tDON concentration and the sampling year (r = −0.158, p < .001). This observation is most probably caused by a significant increase of the urine volume excreted within 24 h (r = 0.345, p < .001). The latter has been analysed in detail by Lermen et al. (2019) for samples of the ESB.

**Fig. 2:**
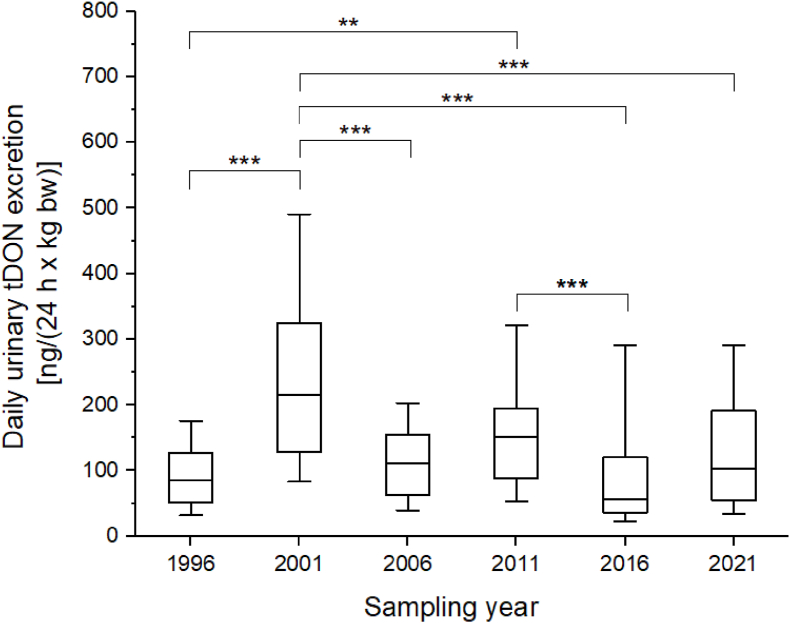
Boxplot of daily urinary tDON excretion normalized to body weight. The boxes and boundaries of the whiskers represent the 25th/75th and 10th/90th percentiles, respectively. The horizontal lines indicate the median values. ***: Significant difference, *p* < .001. **: Significant difference, *p* < .005.

For the daily tDON excretion normalized to body weight, there were significant differences between the sampling years (Kruskal–Wallis test, p < .001). Highest amounts of tDON excreted were observed in 2001 (Fig. 2). The daily tDON excretion was significantly lower in the previous (1996) as well as the following sampling years (2006, 2016 and 2021).

Due to the widespread contamination of cereal-based food with DON, ingestion is the main route of exposure to DON in the general population. To actively decrease the presence in foodstuff, maximum values for DON were set at European level in 2006 (European Commission, 2006). Since a similar level of excreted tDON is observed for all sampling years with exception of 2001, our data do not clearly reflect this policy measure. However, for 2006 and the following sampling years an exceeding of the TDI was not observed in our study population. Reasons for the exceptionally high concentrations measured in 2001 could not be evaluated based on the limited data available.

## 4 Summary and conclusion

To the best of our knowledge, we report the first systematic investigation of the DON exposure for a larger study population (N = 360) over a period of 25 years. tDON could be quantified in 99% of the ESB samples, proving almost all participants were exposed to DON. However, exceedance of the TDI was observed solely for less than 1% of the participants. With exception of 2001, data evaluation revealed an equal magnitude of exposure over all sampling years.

The data give information about the dietary background exposure and show the ubiquitous exposure towards DON. The shown possibility of exceedance of health based guidance values strengthens the importance for further information to identify and assess additional DON exposure like occupational exposure at workplaces e.g. in agriculture, food production and waste management and to evaluate if higher exposed subgroups exist in the population.

## Declaration of competing interest

The authors declare that they have no known competing financial interests or personal relationships that could have appeared to influence the work reported in this paper.
