# Supplementary material for: Human biomonitoring of deoxynivalenol (DON) - Assessment of the exposure of young German adults from 1996 - 2021
Source: Int J Hyg Environ Health. 2023 Jul;252:114198. doi: 10.1016/j.ijheh.2023.114198 (PMC10410250; doi:10.1016/j.ijheh.2023.114198)
Supplement: Multimedia component 1 [file mmc1.docx]

**SUPPLEMENTAL MATERIAL**

Human biomonitoring of deoxynivalenol (DON) -

Assessment of the exposure of young German adults from 1996 – 2021

Andy Schmied, Lennart Marske, Marion Berger, Peter Kujath, Till Weber, Marike Kolossa-Gehring

Content

Table S1: Daily urinary DON excretion.

Table S1

Daily urinary DON excretion. Values are given in µg/24 h.

|  | Sampling year | 1996 | 2001 | 2006 | 2011 | 2016 | 2021 | Σ1996-2021 |
| --- | --- | --- | --- | --- | --- | --- | --- | --- |
| All subjects | N>LLOQ | 60 (100 %) | 59 (98 %) | 60 (100 %) | 60 (100 %) | 60 (100 %) | 58 (97 %) | 357 (99 %) |
|  | AM | 6.86 | 18.4 | 8.17 | 11.7 | 7.87 | 9.57 | 10.4 |
|  | GM | 5.23 | 13.9 | 6.66 | 9.55 | 5.00 | 6.39 | 7.27 |
|  | Median | 6.34 | 14.3 | 7.64 | 9.77 | 4.09 | 6.51 | 7.90 |
|  | P90 | 13.3 | 31.0 | 14.5 | 19.9 | 22.1 | 21.9 | 21.7 |
|  | Range | 0.51-22.7 | 0.38*-90.6 | 0.92-23.3 | 1.08-40.0 | 0.57-40.4 | 0.20*-39.7 | 0.20*-90.6 |
| Males | N>LLOQ | 30 (100 %) | 29 (97 %) | 30 (100 %) | 30 (100 %) | 30 (100 %) | 30 (100 %) | 179 (99 %) |
|  | AM | 8.19 | 22.0 | 9.61 | 14.2 | 8.08 | 13.9 | 12.7 |
|  | GM | 6.1 | 15.0 | 8.13 | 11.4 | 5.18 | 10.6 | 8.79 |
|  | Median | 7.67 | 17.7 | 9.83 | 11.7 | 4.74 | 11.7 | 9.30 |
|  | P90 | 17.9 | 53.000 | 15.2 | 29.9 | 22.1 | 30.9 | 23.5 |
|  | Range | 0.51-22.7 | 0.38*-90.6 | 0.98-23.3 | 3.02-40.0 | 0.57-40.4 | 1.49-39.7 | 0.38*-90.6 |
| Females | N>LLOQ | 30 (100 %) | 30 (100 %) | 30 (100 %) | 30 (100 %) | 30 (100 %) | 28 (93 %) | 178 (99 %) |
|  | AM | 5.52 | 14.8 | 6.74 | 9.15 | 7.67 | 5.19 | 8.17 |
|  | GM | 4.49 | 12.9 | 5.46 | 8.02 | 4.83 | 3.86 | 6.01 |
|  | Median | 4.19 | 12.9 | 5.52 | 8.85 | 3.51 | 4.46 | 6.76 |
|  | P90 | 10.7 | 28.1 | 12.5 | 14.3 | 22.3 | 10.8 | 17.9 |
|  | Range | 1.10-20.0 | 4.15-31.7 | 0.92-15.6 | 1.08-18.4 | 1.00-24.4 | 0.20*-15.8 | 0.20*-31.7 |

N: number of samples; LLOQ: lower limit of quantification; AM: arithmetic mean; GM: geometric mean; P90: 90^th^ percentile; *Volume-based DON concentration of the corresponding 24-h-urine sample is <LLOQ.
